# Mindfulness-Based Interventions for Difficult-to-Treat Major Depressive Disorder: A Systematic Review and Meta-Analysis

**DOI:** 10.1192/j.eurpsy.2025.1374

**Published:** 2025-08-26

**Authors:** I. Borja De Oliveira, L. Robles Rafael, M. Kraide Piedade de Abreu, A. L. Stephany, D. Soler Lopes, M. L. Geremias, F. Wagner, A. V. de Vasconcelos, M. C. Carbajal Tamez, J. Quevedo, M. Teranishi

**Affiliations:** 1 School of Medicine, University of São Paulo, São Paulo; 2 Psychiatry Outpatient Clinic, Integrated Medicine Service, Jacareí; 3 Vila Tesouro Basic Health Unit, São José dos Campos; 4 Foundation of Health and Assistance, Caçapava; 5 São Paulo Association for the Development of Medicine, São Paulo, Brazil; 6 Lancaster University, Lancaster, United Kingdom; 7 University of the Joinville Region, Joinville; 8 School of Medicine, Pontifical Catholic University of Rio Grande do Sul, Porto Alegre; 9 Afya College of Medical Sciences of Santa Inês, Santa Inês, Brazil; 10 Department of Psychiatry and Behavioral Sciences at McGovern Medical School; 11 Center for Interventional Psychiatry, Faillace Department of Psychiatry and Behavioral Sciences, McGovern Medical School, The University of Texas Health Science Center at Houston, Houston, United States; 12 Università degli Studi di Milano, Milan, Italy

## Abstract

**Introduction:**

Major Depressive Disorder (MDD) affects up to 20 million people worldwide over their lifetime. 30% will not attain lasting symptom relief even after multiple treatment attempts. Mindfulness-based interventions (MBIs) have recently been added as adjunctive therapy for MDD. However, their efficacy as adjunctive therapy for difficult-to-treat depression (DTD) remains unclear.

**Objectives:**

This systematic review and meta-analysis sought to evaluate the efficacy of MBIs in treating DTD.

**Methods:**

We conducted a search of MEDLINE, Embase, Web of Science, PsycINFO, Cochrane Central Register of Controlled Trials, the World Health Organization International Clinical Trials Registry Platform, and ClinicalTrials.gov. No restrictions on language or publication date were enforced. We included randomized controlled trials that compared MBIs with usual care or other treatments for unipolar DTD. In this context, DTD refers to the inability to achieve full remission of depressive symptoms despite receiving an adequate course of antidepressant medication. When a sufficient number of studies were available for the outcome analysis, we employed a random-effects model to address the variability between interventions. For outcomes based on a smaller number of studies, we used a fixed-effects model. Additionally, we performed influential and subgroup analyses to investigate the data further. To assess the risk of bias, we utilized the Risk of Bias 2 tool.

**Results:**

Eight studies met our inclusion criteria, comprising 449 participants (mean age = 42.6, predominantly female). The MBIs evaluated included mindfulness-based cognitive therapy, breathing-based meditation, and dialectical behavior therapy with mindfulness components. We found that adjunctive MBIs significantly reduced depressive symptom severity, with an effect size of g = -0.80 (95% CI [-1.32, -0.27], p = 0.0004). Additionally, MBIs caused significant improvements in anxiety (g = -0.57, 95% CI [-0.94, -0.20], p = 0.002) in four studies (n = 126), mindfulness (g = 0.32, 95% CI [0.06, 0.57], p = 0.02) in three studies (n = 243), and psychological well-being (g = 0.66, 95% CI [0.25, 1.07], p = 0.002) in three studies (n = 97).

**Conclusions:**

MBIs demonstrate a substantial benefit for patients with DTD, with a large effect size in reducing depressive symptoms, a medium effect size in improving anxiety and psychological well-being, and a smaller effect size in enhancing mindfulness. Their ability to significantly alleviate depressive symptoms and improve overall mental health supports their integration into treatment plans for DTD. However, this review was limited by the small number of eligible studies, small sample sizes, and high heterogeneity between studies. To better understand the effectiveness of MBIs for DTD, larger clinical studies are needed.

**Disclosure of Interest:**

I. Borja De Oliveira: None Declared, L. Robles Rafael: None Declared, M. Kraide Piedade de Abreu: None Declared, A. Stephany: None Declared, D. Soler Lopes: None Declared, M. Geremias: None Declared, F. Wagner: None Declared, A. de Vasconcelos: None Declared, M. Carbajal Tamez: None Declared, J. Quevedo Shareolder of: Instituto de Neurociencias Dr. Joao Quevedo, Grant / Research support from: LivaNova; and receives copyrights from Artmed Editora, Artmed Panamericana, and Elsevier/Academic Press, Consultant of: EMS, Libbs, and Eurofarma, Speakers bureau of: Myriad Neuroscience and AbbVie., M. Teranishi: None Declared

